# Computer-Aided Decision Support and 3D Models in Pancreatic Cancer Surgery: A Pilot Study

**DOI:** 10.3390/jcm14051567

**Published:** 2025-02-26

**Authors:** Diederik W. M. Rasenberg, Mark Ramaekers, Igor Jacobs, Jon R. Pluyter, Luc J. F. Geurts, Bin Yu, John C. P. van der Ven, Joost Nederend, Ignace H. J. T. de Hingh, Bert A. Bonsing, Alexander L. Vahrmeijer, Erwin van der Harst, Marcel den Dulk, Ronald M. van Dam, Bas Groot Koerkamp, Joris I. Erdmann, Freek Daams, Olivier R. Busch, Marc G. Besselink, Wouter W. te Riele, Rinze Reinhard, Frank Willem Jansen, Jenny Dankelman, J. Sven D. Mieog, Misha D. P. Luyer

**Affiliations:** 1 Faculty of BioMechanical Engineering, Delft University of Technology, 2628 CE Delft, The Netherlands; diederik.rasenberg@philips.com (D.W.M.R.); f.w.jansen@lumc.nl (F.W.J.); j.dankelman@tudelft.nl (J.D.); 2 Department of Experience Design, Philips, 5656 AE Eindhoven, The Netherlands; jon.pluyter@philips.com (J.R.P.); luc.geurts@philips.com (L.J.F.G.); bin.yu@philips.com (B.Y.); 3 Department of Surgery, Catharina Hospital, 5623 EJ Eindhoven, The Netherlands; ignace.d.hingh@catharinaziekenhuis.nl (I.H.J.T.d.H.); misha.luyer@catharinaziekenhuis.nl (M.D.P.L.); 4 Department of Hospital Services & Informatics, Philips Research, 5656 AE Eindhoven, The Netherlands; igor.jacobs@catharinaziekenhuis.nl (I.J.); john.van.der.ven@philips.com (J.C.P.v.d.V.); 5 Department of Radiology, Catharina Hospital, 5623 EJ Eindhoven, The Netherlands; joost.nederend@catharinaziekenhuis.nl; 6 GROW—School for Oncology and Developmental Biology, Maastricht University, 6229 ER Maastricht, The Netherlands; 7 Department of Surgery, Leiden University Medical Centre, 2300 RC Leiden, The Netherlands; b.a.bonsing@lumc.nl (B.A.B.); a.l.vahrmeijer@lumc.nl (A.L.V.); j.s.d.mieog@lumc.nl (J.S.D.M.); 8 Department of Surgery, Maasstad Hospital, 3079 DZ Rotterdam, The Netherlands; harste@maasstadziekenhuis.nl; 9 Department of Surgery, Maastricht University Medical Centre+, 6229 ER Maastricht, The Netherlands; marcel.den.dulk@mumc.nl (M.d.D.); r.van.dam@mumc.nl (R.M.v.D.); 10 Department of Surgery, Erasmus Medical Centre, 3015 GD Rotterdam, The Netherlands; b.grootkoerkamp@erasmusmc.nl; 11 Department of Surgery, Amsterdam University Medical Centers, 1081 HV Amsterdam, The Netherlands; j.i.erdmann@amsterdamumc.nl (J.I.E.); f.daams@amsterdamumc.nl (F.D.); o.r.busch@amsterdamumc.nl (O.R.B.); m.g.besselink@amsterdamumc.nl (M.G.B.); 12 Department of Surgery, St. Antonius Hospital, 3435 CM Nieuwegein, The Netherlands; w.te.riele@antoniusziekenhuis.nl; 13 Department of Radiology, Onze Lieve Vrouwe Gasthuis (loc. West), 1091 AC Amsterdam, The Netherlands; r.reinhard@olvg.nl; 14 Department of Surgery, Leiden University Medical Center, 2333 ZA Leiden, The Netherlands; 15 Department of Electrical Engineering, Eindhoven University of Technology, 5600 MB Eindhoven, The Netherlands

**Keywords:** pancreatic carcinoma, pancreatoduodenectomy, integrated medical imaging workstation, autostereoscopic three-dimensional patient model, computer-aided detection, preoperative planning, vascular involvement

## Abstract

**Background:** Preoperative planning of patients diagnosed with pancreatic head cancer is difficult and requires specific expertise. This pilot study assesses the added value of three-dimensional (3D) patient models and computer-aided detection (CAD) algorithms in determining the resectability of pancreatic head tumors. **Methods:** This study included 14 hepatopancreatobiliary experts from eight hospitals. The participants assessed three radiologically resectable and three radiologically borderline resectable cases in a simulated setting via crossover design. Groups were divided in controls (using a CT scan), a 3D group (using a CT scan and 3D models), and a CAD group (using a CT scan, 3D and CAD). For the perceived fulfillment of preoperative needs, the quality and confidence of clinical decision-making were evaluated. **Results:** A higher perceived ability to determine degrees and the length of tumor–vessel contact was reported in the CAD group compared to controls (*p* = 0.022 and *p* = 0.003, respectively). Lower degrees of tumor–vessel contact were predicted for radiologically borderline resectable tumors in the CAD group compared to controls (*p* = 0.037). Higher confidence levels were observed in predicting the need for vascular resection in the 3D group compared to controls (*p* = 0.033) for all cases combined. **Conclusions:** “CAD (including 3D) improved experts’ perceived ability to accurately assess vessel involvement and supports the development of evolving techniques that may enhance the diagnosis and treatment of pancreatic cancer”.

## 1. Introduction

Pancreatoduodenectomy (PD) is the cornerstone for the surgical treatment of pancreatic cancer. This procedure is technically challenging and accompanied with a high morbidity of 20–30% [1,2,3]. Preoperative planning using radiological imaging is essential to account for the vascular involvement of the tumor and variations in arterial anatomy [2]. Multi-phase (arterial and portal-venous) contrast-enhanced multi-detector computed tomography (MDCT) is standard practice in evaluating pancreatic cancer and the assessment of resectability [3,4]. Standardized resectability criteria are used to tailor the need for neoadjuvant therapy and select patients for a minimal invasive approach [5,6,7]. However, the assessment of resectability based on CT scan remains challenging especially following neoadjuvant treatment [8]. Tumor regression after neoadjuvant treatment is rarely visible on CT, and the amount of vascular involvement tends to be overestimated [9]. Also, the literature shows considerable interobserver variability, even among the most experienced clinicians [10,11]. As such, clinicians are unable to accurately assess tumor resectability, and, based on the promising results of neoadjuvant treatment, it is to be expected that this will become an increasing problem [12]. Therefore, attempts should be made to improve the accuracy of imaging techniques and implement novel technological tools to improve pancreatic cancer assessment.

Computer-aided detection (CAD) algorithms based on artificial intelligence techniques can provide pixel-level segmentations of the pancreatic tumor and surrounding structures using deep learning. Deep learning refers to the use of neural networks with multiple layers, designed to recognize features from input data. In particular, convolutional neural networks (CNNs) have rapidly improved image processing, leading to the development of image-based computer-aided detection (CAD) methods for pancreatic cancer assessment [13]. By offering quantitative metrics on tumor–vessel relationships, CAD can potentially reduce interobserver variability, standardize preoperative assessment, and enhance resectability predictions [14].

Beyond CAD, 3D patient models reconstructed from AI-based segmentations serve a distinct role and offer an additional layer of visualization. Autostereoscopic 3D displays, which provide realistic depth perception without requiring special glasses, allow surgeons and radiologists to better understand complex anatomy and tumor–vessel relation-ships. This combination of CAD and 3D modeling could provide surgeons and radiologists with a better spatial understanding of complex anatomy and tumor–vessel relation-ships, particularly in borderline resectable cases.

The combination of CAD-derived vascular involvement metrics and 3D visualization may further improve tumor assessment, enhance preoperative decision-making, and re-duce variability in resectability assessment. 

This pilot study aims to evaluate the added value of autostereoscopic 3D patient models and CAD-derived vascular involvement metrics for decision support in pancreatic cancer care. 

## 2. Materials and Methods

In this study, 14 participants individually performed preoperative planning for pancreatoduodenectomy in a simulated setting under different study conditions, using an integrated medical imaging workstation. The study was approved by the Internal Committee of Biomedical Experiments (ICBE) of Philips and the Medical Ethical Technical Committee of Catharina Hospital Eindhoven (CZE) under protocol number ICBE-S-000210, with approval granted on 27 May 2021. Informed consent was obtained from each surgeon or radiologist before participation.

### 2.1. Study Participants

This study was conducted with 14 hepatopancreatobiliary experts (thirteen surgeons and one radiologist) from 8 high-volume pancreatic cancer centers in the Netherlands. All participants were males between 36 and 65 years old. Ten participants had 11 or more years of experience as a consultant and four participants had between 6 and 10 years of experience as a consultant. All surgeons performed over 100 open pancreatoduodenectomies (OPDs), and nine surgeons performed over 200 OPDs. Five surgeons had no experience in performing robot-assisted pancreatoduodenectomies (RAPDs), five surgeons performed over 50 RAPDs, and one surgeon performed over 100 RAPDs.

### 2.2. Data Collection

Data of retrospectively collected and de-identified patient cases have been acquired from the Catharina Hospital, Eindhoven, The Netherlands. The data consisted of medical imaging data and relevant clinical information. The clinical information comprised all pre-, intra-, and postoperative information regarding tumor characteristics, vascular involvement, anatomical conformation, and surgical approach coming from the radiology-, pathology-, and surgery reports. Six patient cases with adenocarcinoma in the head of the pancreas that underwent pancreatoduodenectomy in CZE between 2014 and 2018 were included. Three cases were classified as radiologically resectable, and three were classified as borderline resectable based on radiological assessment, according to the DPCG criteria [5].

For the radiological resectable tumors, pathology confirmed there was no vascular involvement, and, in all three cases, a radical tumor resection (R0) was performed. In two radiologically borderline resectable cases, no vascular resection was performed, and an R0 resection status was achieved. In one borderline resectable case, a vascular resection (R1) was performed. The microscopic irradicality was not on the venous side. All patients underwent open pancreatoduodenectomy, and no patients had received neoadjuvant therapy.

### 2.3. Segmentation of the Tumor and Surrounding Blood Vessels

**Figure 1:**
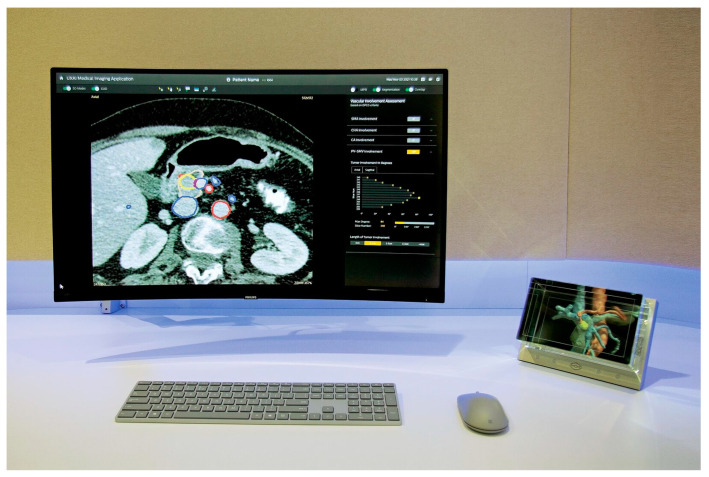
The integrated medical imaging workstation. The integrated medical imaging workstation consisting of a DICOM viewer showing the CT scan, segmentations, and the Looking Glass holographic display. The segmentations including information on tumor vessel contact were reconstructed and displayed on the Looking Glass holographic display.

An arterial and/or a portal-venous-phase CT scan was acquired for each patient. Relevant anatomical structures (tumor, pancreas, aorta, superior mesenteric artery, celiac axis, common hepatic artery, splenic artery, gastroduodenal artery, vena cava, vena porta, superior mesenteric vein, inferior mesenteric vein, splenic vein, and aberrant arteries) were manually annotated by a PhD student (Medical Doctor) and supervised by an expert abdominal radiologist from CZE using IntelliSpace Portal software (version 12.1.5) (Philips, Eindhoven, the Netherlands) [15]. Annotations simulated CAD-generated segmentations and enabled early collection of clinical feedback on to-be-developed AI models. The pixel-level segmentations were exported for 3D model reconstruction as visualization tool kit (VTK) files. CT scans were uploaded as Digital Imaging and Communication in Medicine (DICOM) files to a locally hosted DICOM server (Orthanc) [16]. The degrees of involvement and the length of tumor–vessel trajectories were quantified in Matlab and presented as numerical information (MathWorks, Natick, MA, USA) [17]. The segmentations including information on tumor vessel contact were reconstructed as a three-dimensional model into Unity (Unity Technologies, San Francisco, CA, USA) and displayed on the Looking Glass holographic display (Looking Glass Factory, Brooklyn, NY, USA) alongside a DICOM viewer showing the CT scans, segmentations, and tumor vessel contact information (Figure 1) [18,19,20]. The holographic display provides realistic depth perception of patient anatomy without the need to wear headgear.

### 2.4. Study Design

The simulated surgical planning sessions consisted of three phases, starting with the pre-test questionnaire, followed by the simulated surgical planning, and ending with a post-test questionnaire. The pre-test questionnaire captured information regarding surgical experience and the extent to which participants perceived that their clinical needs were fulfilled by technology in current practice (Likert scale). The needs were divided into five categories: 1. tumor detection/localization, 2. preoperative tumor assessment, 3. preoperative vascular involvement assessment, 4. preoperative anatomical understanding, and 5. intraoperative understanding (Supplementary Material S1).

Subsequently, all participants performed simulated surgical planning on six cases via a crossover design in which each participant assessed two different case types (one radiological resectable and one radiological borderline resectable). Resectability was classified according to the DPCG criteria for resectability [5].

In the control group, experts used only a regular CT image viewer with basic image manipulation functionalities (zooming, panning, windowing, and multi-planar reconstruction). In the 3D group, experts assessed the pancreas tumor using the CT image viewer, segmentations outlining anatomical structures, and autostereoscopic 3D patient models. In the CAD group, pancreatic tumors were assessed using the CT image viewer. The evaluation included segmentations outlining anatomical structures, autostereoscopic 3D patient models, and CAD-derived metrics regarding vascular involvement. Numeric information, such as the estimated number of degrees and length of vessel contact, was also analyzed.

Participants started with the control assessment (CT group), followed by the 3D assessment (3D group), and finally the CAD assessment (CAD group) technology. For each patient case, participants were asked to determine the degrees of tumor–vessel contact, predict tumor resectability, predict whether vascular resection was needed, and identify potential vascular variations. Additionally, the participants’ confidence regarding resectability and vascular resection assessment was determined (Supplementary Material S2). Lastly, participants filled in a post-test questionnaire to assess the extent to which participants perceived that their clinical needs were fulfilled by the proposed 3D and CAD technology and experience working with CAD (Supplementary Material S3).

### 2.5. Outcome Measures

Primary outcome of this study was the perceived fulfillment of clinical needs around key aspects of the surgical planning before and after the study. Secondary outcomes of this study were the degrees of tumor contact, the prediction accuracy, and the confidence regarding vascular involvement prediction.

The perceived fulfillment of clinical needs was measured on a Likert scale (1 = strongly disagree and 5 = strongly agree). The degrees of tumor contact were expressed as an ordinal variable (no contact, <90° contact with PV-SMV, between 90 and 180° contact with PV-SMV, >180° contact with PV-SMV, and cannot determine) for each study condition. Predictions were considered correct if the answer that is given by the participant corresponded to the actual ground truth based on the pathology- and surgical report.

The prediction accuracy (*number of correct predictions*)/(*total number of predictions*) was defined for the following three decisions: 1. tumor resectability, 2. need for vascular resection, and 3. presence of an anatomical variation. Confidence regarding resectability assessment and need for vascular resection predictions was expressed on a 1–10 scale (1 = low and 10 = high).

### 2.6. Statistical Analysis

Data regarding perceived fulfillment of clinical needs, the degrees of tumor–vessel contact, and the level of confidence were imported into Matlab versionR2022b (MathWorks, Natick, MA, USA) for statistical analysis [16]. The ANOVA Kruskal–Wallis test was performed to analyze the perceived fulfillment of clinical needs, the degrees of tumor contact decisions, and confidence levels. This statistical test is used since the sample sizes are relatively small, and the outcomes did not have a normal distribution [21]. The confidence and need fulfillment were expressed as the median and the interquartile ranges (IQRs). Multiple comparison tests were performed using the Kruskal–Wallis results to determine whether the mean ranks of the conditions are significantly different. A *p*-value of <0.05 was considered statistically significant.

## 3. Results

### 3.1. Perceived Fulfillment of Needs

**Figure 2:**
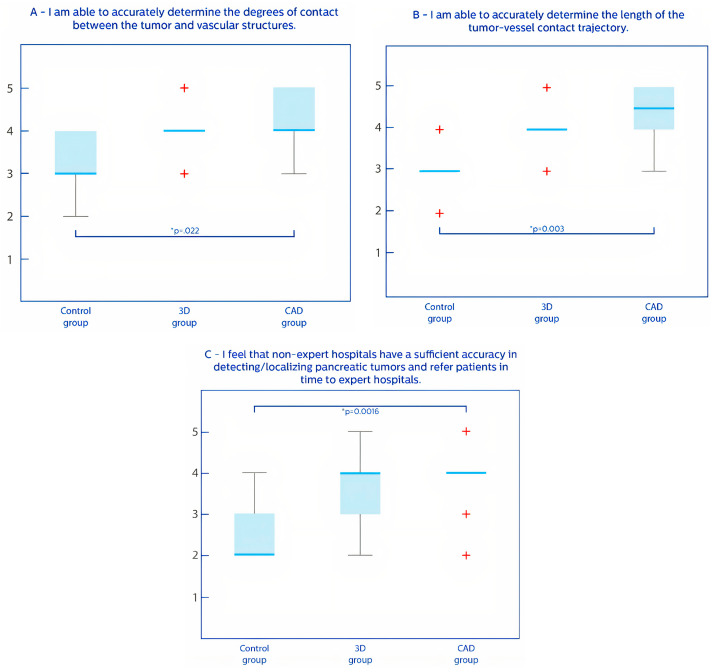
Box plots of perceived fulfillment of clinical needs. Fulfillment of clinical needs were scored on a Likert scale (1 = completely disagree and 5 = completely agree). Blue lines = medians; light blue boxes = 25th and 75th percentile; red crosses = outlier values; grey line = range of values. * *p* < 0.05.

The perceived fulfillment of clinical needs, reflecting the opinion of the participants on 3D and CAD, showed no significant differences between current practice (pre-test) and the control group (post-test), thereby validating the control group (Figure 2). The participants indicated that CAD-derived metrics would improve their ability to accurately determine the degrees of vessel contact compared to the control group (*p* = 0.022) (Figure 2A). Additionally, the participants indicated that CAD-derived metrics would also increase their ability to accurately assess the length of the tumor–vessel trajectory compared to the control group (*p* = 0.003) (Figure 2B). Additionally, the participants indicated that, in their opinion, non-experts would benefit from 3D and CAD since this would improve the ability to detect and localize pancreatic tumors more accurately (*p* = 0.002) (Figure 2C).

### 3.2. Prediction Accuracy

The participants correctly predicted tumor resectability and the need for vascular resection for all radiologically resectable cases. For the radiologically borderline resectable cases, five participants correctly predicted resectability in the control group compared to seven in the 3D group and eight in the CAD group (Table 1). This was not significantly different between groups. Remarkably, two participants reported one radiologically borderline resectable case as irresectable in the control group, while, in fact, the tumor was resectable according to the surgical report, and an R0 resection margin was achieved. All participants correctly did not report irresectable tumors in the 3D group or CAD group for all cases.

**Figure 3:**
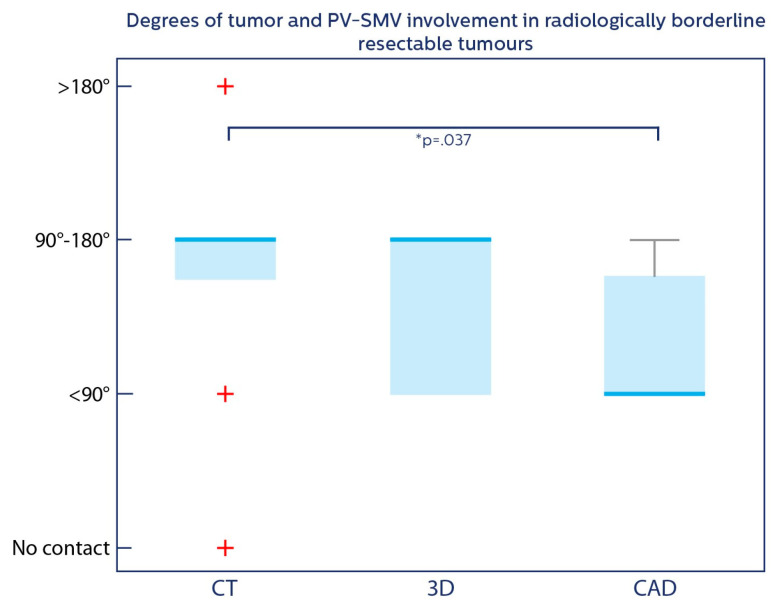
Box plot regarding degrees of tumor and PV–SMV involvement. Box plot regarding the degrees of contact between tumor and portal vein/superior mesenteric vein in radiologically borderline resectable tumors. Blue lines = medians; blue boxes = 25th and 75th percentile; red crosses = outlier values; grey line = range of values. * *p*–value < 0.05.

Seven participants correctly predicted whether a vascular resection was needed in the control group compared to five in the 3D group and five in the CAD group (Table 1). Six participants in the control group indicated that they were not able to determine if a vascular resection was needed compared with two participants in the 3D group and three participants in the CAD group. The participants predicted lower degrees of tumor–vessel contact for radiologically borderline resectable cases when supported by 3D models and CAD-derived metrics compared to regular radiological assessment (*p* = 0.037) (Figure 3).

Patients with aberrant vascular anatomy were correctly identified in 22 of 27 evaluations in the control group, 25 of 28 in the 3D group, and 26 of 29 in the CAD group (Supplementary Material S5). Among patients with arterial variations, 4 of 18 variations were missed in the control group, compared with 2 of 19 in the 3D group and 3 of 19 in the CAD group. These differences were not statistically significant between groups.

### 3.3. Confidence Levels

In general, confidence levels for resectability assessment and the need for vascular resection were significantly lower in the radiologically borderline resectable cases compared to the radiologically resectable cases in all groups (Supplementary Material S6). This indicates that case complexity corresponded with the confidence levels of the participants regarding vascular involvement assessment. Significantly higher confidence levels were observed in predicting the need for vascular resection in the 3D group compared to the control group (*p* = 0.033) for all cases combined (Table 2). No significant differences were seen in confidence levels of resectability prediction between all three groups.

## 4. Discussion

This study highlights the distinct yet complementary roles of CAD and 3D models in preoperative pancreatic cancer assessment. CAD-derived metrics improved the perceived accuracy of tumor–vessel contact assessment and reduced vascular involvement overestimation on CT, reinforcing its value in quantitative evaluation. Additionally, 3D modeling combined with CAD metrics enhanced tumor localization and vascular assessment, which may be particularly beneficial for non-experts.

In this study, considerable interobserver variability was observed in determining resectability of pancreatic cancer on CT. Vascular involvement prediction in the control group was overestimated compared with the clinical outcome, which is in line with previous reports [22,23]. As shown in Figure 3, CAD reduced this overestimation of degrees of vascular involvement, and the participants consistently reported less vascular involvement in borderline resectable cases when supported by the 3D models and CAD-derived metrics. On one hand, this may indicate that experts supported by 3D combined with CAD better identify the tumor–vessel relation; however, it may also mean that expert clinicians tend to follow the 3D visualization and CAD quantification without being questioned. It is, therefore, of crucial importance that the 3D model and CAD-derived metrics are highly accurate to prevent the risk of overtrust in 3D and CAD. Over time, unexpected findings or repeated inaccurate predictions can result in undertrust, which may hamper implementation. Providing transparency on which data the algorithm is trained and validated on and communicating the model performance and uncertainty of the CAD segmentations and 3D models may be important to achieve appropriate trust [24].

Interestingly, 3D patient models combined with CAD-derived metrics did not result in a more accurate prediction regarding the need for vascular resection in this study. Notably, two participants incorrectly classified a resectable tumor as irresectable in the control group, a misclassification that did not occur in the 3D or CAD groups. The participants consistently reported less vascular involvement when supported by the 3D models and CAD, making the participants less likely to predict the need for vascular resection. This indicates that these tools may help prevent the overestimation of vascular involvement, which is a known limitation of CT-based assessments. Also, the need for a vascular resection can be surgeon-dependent. Since predictions were compared to actual ground truth based on the pathology- and surgical report, this may explain a lower prediction accuracy. Additionally, the accuracy of CAD may be further improved by the optimization of algorithms using artificial intelligence (AI). Retrospective studies show that AI may improve tumor detection and may also be used to better visualize contact of tumor with surrounding tissue [24]. Although clinicians demonstrate acceptable sensitivity measures in predicting vascular involvement, AI has the potential to achieve a better and personalized treatment plan for each patient. This may be especially important in the era of neoadjuvant therapy since the sensitivity of CT in predicting vascular involvement drops from 80% to 50% after neoadjuvant treatment [23]. Based on the promising results of neoadjuvant treatment, it is to be expected that the accurate assessment of vascular involvement will become increasingly important. However, future studies are needed to show the additive effect of these algorithms to current practice.

Unexpectedly, 3D patient models and CAD-derived metrics did not result in higher confidence regarding the prediction of resectability. Case complexity and the distinguishability of the tumor were the main determinants for a certain level of confidence according to the participants. This might explain comparable confidence levels across groups. Nevertheless, the 3D patient models provided clinicians with significantly more confidence in predicting whether a vascular resection was needed to achieve an R0 resection margin for all cases combined (*p* = 0.033). Although 3D patient models and CAD did not result in a more accurate prediction regarding the need for vascular resection, the participants indicated that CAD and 3D models provided a realistic and intuitive understanding of pancreatic tumors that were similar as they encountered during surgery. The ability to see what a case will look like is extremely important for safe pancreatic surgery, and 3D models could help in acquiring confidence to move through complex resections. This may have resulted in higher levels of confidence and emphasizes that while 3D and CAD models may refine vascular assessment, their role in overall resectability prediction requires further investigation.

In clinical practice, CAD and 3D models offer distinct advantages in clinical settings. CAD provides automated tumor–vessel quantification, improving diagnostic accuracy and reducing observer variability, but it may misinterpret complex anatomical variations. Three-dimensional models enhance spatial understanding, aiding surgical planning by clearly visualizing tumor location and its relationship to surrounding structures. However, their creation is time-consuming and dependent on imaging quality. Therefore, while each tool has its strengths, their selective application should be carefully considered based on the specific clinical scenario. Integrating both CAD and 3D modeling may provide a more comprehensive approach, leveraging the quantitative analysis of CAD and the spatial visualization of 3D models to improve surgical outcomes in pancreatic cancer treatment.

This pilot study has several limitations. First, some participants mentioned a relatively low CT resolution that could make tumor localization less reliable and might have influenced their confidence in decisions. The prototype included only one contrast phase of the CT scan, while in clinical practice, clinicians typically use two phases to assess the patient. Decisions and the confidence levels might have been influenced by the absence of the arterial or portal-venous phase. Nevertheless, when compared with usual care, the CT group was not different. Lastly, segmentations were annotated manually to simulate the CAD output. Although realistically simulated, this could have influenced the trust and confidence of the participants in the segmentations. Nonetheless, the realistic simulation of AI proved to be a useful approach to meaningfully engage clinicians in an early phase of development. Therefore, this pilot study gained important insights into AI model requirements and clinician–AI interaction to achieve optimal decisions, facilitate seamless workflow integration, and achieve appropriate trust.

## 5. Conclusions

In conclusion, this study demonstrates that computer-aided detection (CAD) and 3D modeling play distinct yet complementary roles in the preoperative assessment of pancreatic head cancer. CAD provides objective, quantitative metrics of tumor–vessel involvement, enhancing assessment standardization and potentially reducing interobserver variability. Three-dimensional models offer improved spatial understanding, particularly in borderline resectable cases, thereby increasing clinician confidence in decision-making regarding vascular involvement. Integrating both technologies into clinical workflows may lead to more informed decision-making, optimized preoperative planning, and improved patient outcomes. Future studies should focus on assessing appropriate trust in these tools to achieve optimal clinical decision-making.

## Figures and Tables

**Table 1:** Prediction accuracy of vascular involvement assessment.

	CT Group	3D Group	CAD Group
Category	Correct (n)/Total (n)	Correct (n)/Total (n)	Correct (n)/Total (n)
Resectability			
All cases combined	18/27	22/28	22/29
Radiologically resectable	13/13	15/15	14/14
Radiologically borderline resectable	5/14	7/13	8/15
**Vascular resection**			
All cases combined	19/27	19/28	18/29
Radiologically resectable	12/13	14/15	13/14
Radiologically borderline resectable	7/14	5/13	5/15

Prediction accuracy was calculated by the formula (number of correct predictions)/(number of predictions). Correct (n) = correct predictions; total (n) = total number of predictions. The superscript reports the number of times a participant could not determine whether vascular resection was needed.

**Table 2:** Levels of confidence.

Category	CT Group Median [25th and 75th Percentile]	3D Group Median [25th and 75th Percentile]	CAD Group Median [25th and 75th Percentile]	CT vs. 3D *p*-Value	CT vs. CAD *p*-Value	3D vs. CAD *p*-Value
All cases combined						
Resectability	8 [6.25–9]	8 [8–10]	8 [7.75–9]	0.17	0.29	0.94
Vascular resection	7 [6–8]	9 [7–9]	8 [6.75–9]	0.033 *	0.26	0.59

Median, 25th and 75th percentiles were calculated with the Kruskal–Wallis statistical tests. *p*-values for comparing different groups were calculated by performing multi-comparison testing. * *p*-value < 0.05.

## Data Availability

The data presented in this study are available on request from the corresponding author.

## Supplementary Materials

The following supporting information can be downloaded at https://www.mdpi.com/article/10.3390/jcm14051567/s1, Supplementary Material S1—Pre-test questionnaire study of 3D and CAD in pancreatic cancer; Supplementary Material S2—Study of 3D and CAD in pancreatic cancer; Supplementary Material S3—Post-test questionnaire study of 3D and CAD in pancreatic cancer; Supplementary Material S4—Perceived Need Fulfillment; Supplementary Material S5—Prediction accuracy for determining an anatomical variation; Supplementary Material S6—Confidence levels.
