# Supplementary material for: Computer-Aided Decision Support and 3D Models in Pancreatic Cancer Surgery: A Pilot Study
Source: J Clin Med. 2025 Feb 26;14(5):1567. doi: 10.3390/jcm14051567 (PMC11899912; doi:10.3390/jcm14051567)
Supplement: Supplementary file 1 [file jcm-14-01567-s001.zip › jcm-3332999-supplementary.pdf]

Supplementary material - Computer-Aided Decision Support and 3D patient modelling in Pancreatic Cancer: A Multi-Centre Pilot Study.

Supplementary material S1 - Pre-test questionnaire study 3D and CAD in pancreatic cancer

Participant: ________________

Part A: General information

1. What is your age?

□ < 25 years □ 26-35 years □ 36-45years □ 46-55 years □ 56-65 years □ 66+ years

2. Are you color vision deficient (kleurenblind)?

□ Yes, type of deficiency ______________________ □ No

3. Are you left- or right-handed?

□ Left □ Right

4. What is your medical specialism: ______________________

5. How many years of experience as medical specialist do you have?

□ < 5 years □ 6-10 years □ 11-15 years □ 16-20 years □ >20 years

6. How many open and robot- assisted pancreaticoduodenectomies do you have performed in your career?

- Open pancreaticoduodenectomies (OPD): __________ times
- Robot-assisted pancreaticoduodenectomies (RAPD): __________ times

7. What imaging modalities do you currently use to evaluate the patient during the preoperative phase of pancreaticoduodenectomies?

…

8a. In the assessment of tumor resectability based on vascular involvement, in what percentage of cases are you NOT confident about the decision?

**1**	**2**	**3**	**4**	**5**
<20%	<40%	40-60%	>60%	>80%

8b. Main factors contributing to a lower confidence are:

…

9. In the assessment of pancreatic cancer resectability based on vascular involvement, how frequently do you feel the need for more information?

**1**	**2**	**3**	**4**	**5**
Never	Rarely	Sometimes	Often	Always
Namely:				

10. Among my peers, I am usually the first to try out new information technologies:

**1**	**2**	**3**	**4**	**5**
Strongly disagree	Disagree	Neutral	Agree	Strongly agree

Three-dimensional visualization techniques

11. Do you use or have used 3D stereoscopic displays?

In work / clinical setting

**1**	**2**	**3**	**4**	**5**
Never	Rarely	Sometimes	Often	Always

In personal setting (e.g. in gaming)

**1**	**2**	**3**	**4**	**5**
Never	Rarely	Sometimes	Often	Always

12. Do you use or have used Virtual Reality or Augmented Reality display techniques?

In work / clinical setting

**1**<br>Never	**2**<br>Rarely	**3**<br>Sometimes	**4**<br>Often	**5**<br>Always

In personal setting (e.g. in gaming)

**1**<br>Never	**2**<br>Rarely	**3**<br>Sometimes	**4**<br>Often	**5**<br>Always

Computer Aided Detection and Diagnosis

13. In your daily clinical work, how frequently do you work with Computer Aided <u>Detection*</u> tools?

**1**<br>Never	**2**<br>Rarely	**3**<br>Sometimes	**4**<br>Often	**5**<br>Always

14. How frequently do you trust the output given by Computer Aided <u>Detection*</u> tools?

**1**<br>Never	**2**<br>Rarely	**3**<br>Sometimes	**4**<br>Often	**5**<br>Always

15. What is your general opinion about Computer Aided Detection* tools?

…

** Computer Aided Detection tools focus on highlighting, segmenting, or measuring potentially interesting anatomical structures or areas (e.g., nodule detection). Interpretation is done by the radiologist (e.g., tumor is likely malignant).*

16. In your daily clinical work, how frequently do you work with Computer Aided <u>Diagnosis**</u> tools?

**1**<br>Never	**2**<br>Rarely	**3**<br>Sometimes	**4**<br>Often	**5**<br>Always

17. How frequently do you trust the output given by Computer Aided <u>Diagnosis**</u> tools?

**1**<br>Never	**2**<br>Rarely	**3**<br>Sometimes	**4**<br>Often	**5**<br>Always

18. What is your general opinion about Computer Aided Diagnosis* tools?

…

** Computer Aided Detection tools focus on highlighting, segmenting, or measuring potentially interesting anatomical structures or areas (e.g., nodule detection). Interpretation is done by the radiologist (e.g., tumor is likely malignant).*

Part B: Pre-test questionnaire to measure clinical needs

Philips has explored and identified in collaboration with Catharina Hospital Eindhoven different clinical needs of HPB-surgeons during the preoperative planning of pancreaticoduodenectomies.

In this questionnaire, we want to explore how these identified clinical needs are fulfilled in **current practice** with the **currently available imaging modalities**. If clinical needs of HPB surgeons are missing in this list, you can state these needs at the end of the questionnaire.

If a question is **not applicable**, please do not fill in a score.

Tumor detection and localization

	**By means of the currently available medical imaging modalities…**	**Strongly disagree**	**Disagree**	**Neither agree nor disagree**	**Agree**	**Strongly agree**
1	I am able to accurately detect/localize pancreatic tumors.	1	2	3	4	5
2	I feel that non-expert hospitals have a sufficient accuracy in detecting/localizing pancreatic tumors and refer patients in time to expert hospitals.	1	2	3	4	5
3	I am able to detect metastases (in liver, lymph node, and other organs).	1	2	3	4	5
Comments:						

Preoperative tumor assessment

	**By means of the currently available medical imaging modalities…**	**Strongly disagree**	**Disagree**	**Neither agree nor disagree**	**Agree**	**Strongly agree**
4	I am able to discriminate between types of abnormalities (carcinoma, benign tumor, pancreatitis).	1	2	3	4	5
5	I am able to discriminate between tumor, inflammatory and healthy tissue before neoadjuvant therapy.	1	2	3	4	5
6	I am able to discriminate between tumor, inflammatory, fibrotic, treated and healthy tissue after neoadjuvant therapy.	1	2	3	4	5
Comments:						

Preoperative vascular involvement assessment

	By means of the currently available medical imaging modalities…	Strongly disagree	Disagree	Neither agree nor disagree	Agree	Strongly agree
7	I am able to accurately determine the degrees of contact between the tumor and vascular structures.	1	2	3	4	5
8	I am able to accurately determine the length of the tumor-vessel contact trajectory.	1	2	3	4	5
9	I am able to accurately determine the extend of vascular ingrowth of the tumor in the relevant vessels.	1	2	3	4	5
Comments:						

Preoperative anatomical understanding

	By means of the currently available medical imaging modalities…	Strongly disagree	Disagree	Neither agree nor disagree	Agree	Strongly agree
10	I am able to accurately identify/localize and understand the spatial conformation of the anatomy (e.g. bifurcation of jejunal branch).	1	2	3	4	5
11	I am able to identify/localize potential anatomical variations	1	2	3	4	5
12	I am able to determine if I need I do a vascular resection and how I need to reconstruct the vessel.	1	2	3	4	5
13	I am able to identify (patient specific) anatomical waypoints/landmarks that affirm my surgical approach.	1	2	3	4	5
Comments:						

Intraoperative anatomical understanding

	By means of the currently available medical imaging modalities…	Strongly disagree	Disagree	Neither agree nor disagree	Agree	Strongly agree
14	I am able to create a common understanding within the surgical team of the patient specific anatomy and the surgical plan.	1	2	3	4	5
15	I have a good view of the tumor and the surrounding anatomical structures during open surgery.	1	2	3	4	5
16	I have a good view of the tumor and the surrounding anatomical structures during minimally invasive (robot) surgery.	1	2	3	4	5
Comments:						

Extra clinical need during the preoperative planning

	By means of the currently available medical imaging modalities…
17	[extra clinical need] …
18	[extra clinical need] …
19	[extra clinical need] …

Supplementary material S2 –study 3D and CAD in pancreatic cancer

PC: **Patient code:** **Group:** 1 (CT) / 2 (3D) / 3 (CAD)

**1)** Vascular involvement?	○ No contact ○ Yes, contact ○ Yes, ingrowth ○ Not to be determined
**2A)** Number of degrees involvement?<br>**2B)** Length of the involved trajectory?<br>**2C)** Reduction of vessel lumen?<br>(Choose: 0% / <50% / >50% / 100%)	**SMA:** ………. degrees ………. mm ………. % **CHA:** ………. degrees ………. mm ………. % **CA:** ………. degrees ………. mm ………. % **PV-SMV:** ………. degrees ………. Mm ………. %
**3)** Anatomical variant?	○ No, normal ○ Yes, …………………………..
**4)** CA and/or SMA accessible?	○ Yes, both ○ Stenoses CA ○ Not to be determined ○ Stenosis SMA
**5A) Resectability** (according DPCG criteria)?	○ Resectable ○ Borderline resectable ○ Irresectable
**5B)** Confidence level resectability (1-10)?	Low 1 2 3 4 5 6 7 8 9 10 High
**6A) Neoadjuvant therapy**?	○ Yes ○ No
**6B)** Confidence level neoadjuvant therapy (1-10)?	Low 1 2 3 4 5 6 7 8 9 10 High
**7A) Vascular resection needed?**	○ Yes ○ No ○ Not to be determined
**7B)** Confidence level vascular resection (1-10)?	Low 1 2 3 4 5 6 7 8 9 10 High
**8A) Surgical technique?**	○ OPD ○ RAPD
**8B)** Confidence level surgical technique (1-10)?	Low 1 2 3 4 5 6 7 8 9 10 High
**9) Seen over the course of the complete case;** Is there any aspect of the displayed information that makes you more or less confident? If so, what and why?	○ More ○ Less ○ Not applicable<br>What information?<br>Why?
**10)** Comments?	

Supplementary material S3 – Post-test questionnaire study 3D and CAD in pancreatic cancer

Part A: Post-test questionnaire to measure clinical needs

We want to explore in this questionnaire how the identified clinical needs are fulfilled (Likert scale) in the situation when your preoperative planning would be supported by means of this prototype.

You have evaluated and assessed two cases of pancreatic cancer per user-test condition, namely the CT-, the 3D- and the Quantifications- (in short CAD) group. For each question it is the intention to score the need fulfillment per user-test condition.

If a question is **not applicable**, please do not fill in a score.

Participant: ____________________

Tumor detection and localization

	With the help of this prototype…		Strongly disagree	Disagree	Neither agree nor disagree	Agree	Strongly agree
1	I am able to accurately detect/localize pancreatic tumors.	**CT**	1	2	3	4	5
		**CT+3D**	1	2	3	4	5
		**CT+3D+CAD**	1	2	3	4	5
2	I feel that non-expert hospitals have a sufficient accuracy in detecting/localizing pancreatic tumors and refer patients in time to expert hospitals.	**CT**	1	2	3	4	5
		**CT+3D**	1	2	3	4	5
		**CT+3D+CAD**	1	2	3	4	5
3	I am able to detect metastases (in liver, lymph node, and other organs).	**CT**	1	2	3	4	5
		**CT+3D**	1	2	3	4	5
		**CT+3D+CAD**	1	2	3	4	5
Comments:							

Preoperative vascular involvement assessment

	With the help of this prototype…		Strongly disagree	Disagree	Neither agree nor disagree	Agree	Strongly agree
4	I am able to discriminate between types of abnormalities (carcinoma, benign tumor, pancreatitis).	CT	1	2	3	4	5
		CT+3D	1	2	3	4	5
		CT+3D+CAD	1	2	3	4	5
5	I am able to discriminate between tumor, inflammatory and healthy tissue before neoadjuvant therapy.	CT	1	2	3	4	5
		CT+3D	1	2	3	4	5
		CT+3D+CAD	1	2	3	4	5
6	I am able to discriminate between tumor, inflammatory, fibrotic, treated and healthy tissue after neoadjuvant therapy.	CT	1	2	3	4	5
		CT+3D	1	2	3	4	5
		CT+3D+CAD	1	2	3	4	5
Comments:							

Preoperative anatomical understanding

	With the help of this prototype…		Strongly disagree	Disagree	Neither agree nor disagree	Agree	Strongly agree
7	I am able to accurately determine the degrees of contact between the tumor and vascular structures.	CT	1	2	3	4	5
		CT+3D	1	2	3	4	5
		CT+3D+CAD	1	2	3	4	5
8	I am able to accurately determine the length of the tumor-vessel contact trajectory.	CT	1	2	3	4	5
		CT+3D	1	2	3	4	5
		CT+3D+CAD	1	2	3	4	5
9	I am able to accurately determine the extend of vascular ingrowth of the tumor in the relevant vessels.	CT	1	2	3	4	5
		CT+3D	1	2	3	4	5
		CT+3D+CAD	1	2	3	4	5
Comments:							

Intraoperative anatomical understanding

	With the help of this prototype…		Strongly disagree	Disagree	Neither agree nor disagree	Agree	Strongly agree
10	I am able to accurately identify/localize and understand the spatial conformation of the anatomy (e.g. bifurcation of jejunal branch).	CT	1	2	3	4	5
		CT+3D	1	2	3	4	5
		CT+3D+CAD	1	2	3	4	5
11	I am able to identify/localize potential anatomical variations	CT	1	2	3	4	5
		CT+3D	1	2	3	4	5
		CT+3D+CAD	1	2	3	4	5
12	I am able to determine if I need I do a vascular resection and how I need to reconstruct the vessel.	CT	1	2	3	4	5
		CT+3D	1	2	3	4	5
		CT+3D+CAD	1	2	3	4	5
13	I am able to identify (patient specific) anatomical waypoints/landmarks that affirm my surgical approach.	CT	1	2	3	4	5
		CT+3D	1	2	3	4	5
		CT+3D+CAD	1	2	3	4	5
Comments:							

Intraoperative anatomical understanding

	With the help of this prototype…		Strongly disagree	Disagree	Neither agree nor disagree	Agree	Strongly agree
14	I am able to create a common understanding within the surgical team of the patient specific anatomy and the surgical plan.	CT	1	2	3	4	5
		CT+3D	1	2	3	4	5
		CT+3D+CAD	1	2	3	4	5
15	I have a good view of the tumor and the surrounding anatomical structures during open surgery.	CT	1	2	3	4	5
		CT+3D	1	2	3	4	5
		CT+3D+CAD	1	2	3	4	5
16	I have a good view of the tumor and the surrounding anatomical structures during minimally invasive (robot) surgery.	CT	1	2	3	4	5
		CT+3D	1	2	3	4	5
		CT+3D+CAD	1	2	3	4	5
Comments:							

Part B: CAD evaluation

To answer the questions in this section, the participants must take only the quantifications group into consideration.

1. In exploring and assessing the two pancreatic cases in the quantifications group, how frequently did you understand why* the CAD provided these suggestions?

** e.g., what it based the recommendations on, how it interpreted the findings, etc.*

**1**<br>Never	**2**<br>Rarely	**3**<br>Sometimes	**4**<br>Often	**5**<br>Always
Why (not)?				
What do you think can help you to better understand why the CAD provided these recommendations?				

2. In exploring and assessing the two pancreatic cases in the quantifications group, how frequently did you trust the recommendations given by the CAD?

**1**<br>Never	**2**<br>Rarely	**3**<br>Sometimes	**4**<br>Often	**5**<br>Always
Why (not)?				
What do you think can help you to better trust the CAD recommendations?				

3. In exploring and assessing the two pancreatic cases in the quantifications group, how frequently was there any conflict between your judgement and the CAD recommendation?

**1**<br>Never	**2**<br>Rarely	**3**<br>Sometimes	**4**<br>Often	**5**<br>Always
If so, how did you think and feel about this conflict?				
Why (not)?				

4. In exploring and assessing the two pancreatic cases in the quantifications group, how frequently did the CAD change your mind about the conclusion?

**1**<br>Yes	**2**<br>No
If yes, how is it different?	
If yes, what caused this change?	

5. After having explored and assessed the two pancreatic cases in the quantifications group, did your general opinion on CAD change?

**1**<br>Yes	**2**<br>No
If yes, how is it different?	
If yes, what caused this change?	
If yes, how did this changed our opinion on future CAD-suggestion(s)?	

Supplementary material S4 – Perceived Need Fulfilment

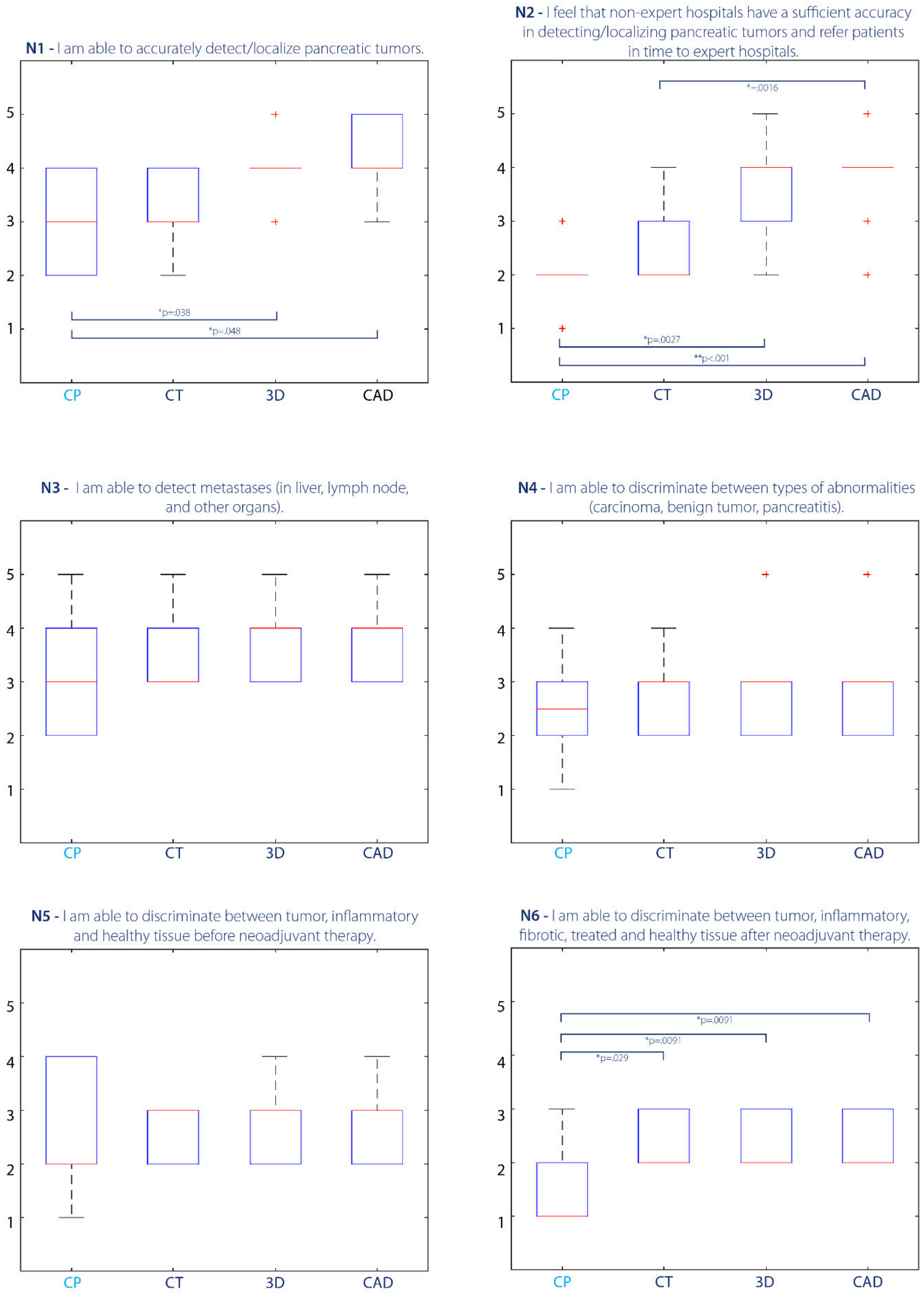

Figure S1. Box plots regarding perceived fulfilment of clinical needs (Likert scale); CP = current practice (pre-test); CT = computed tomography group; 3D = 3D group; CAD = Quantifications group. Red lines = medians; blue boxes = 25th and 75th percentile; red crosses = outlier values; dotted black line = range of values. *p < .05; **p<.001.

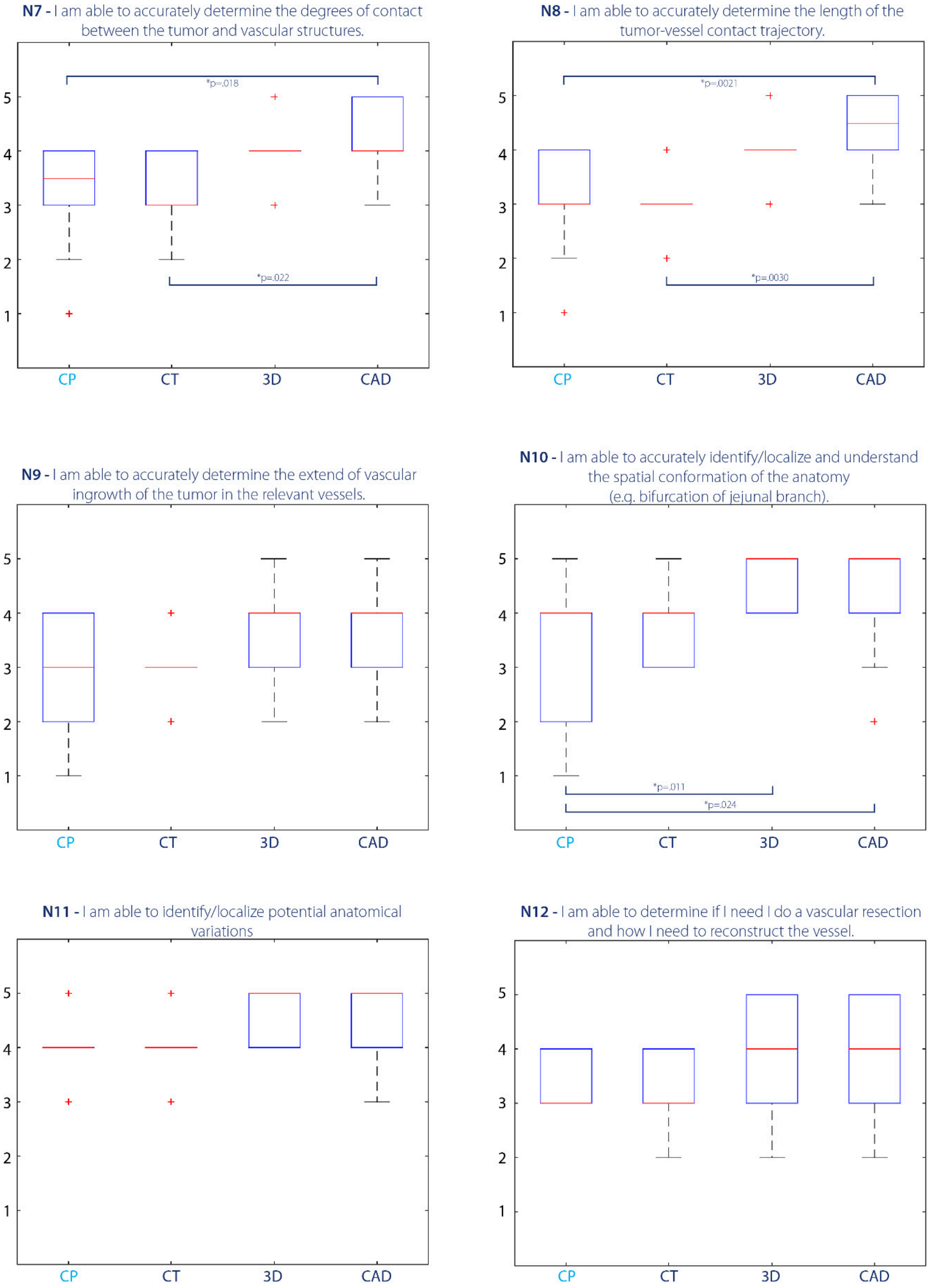

Figure S2. Box plots regarding perceived fulfilment of clinical needs (Likert scale); CP = current practice (pre-test); CT = computed tomography group; 3D = 3D group; CAD = Quantifications group. Red lines = medians; blue boxes = 25th and 75th percentile; red crosses = outlier values; dotted black line = range of values. *p < .05; **p<.001.

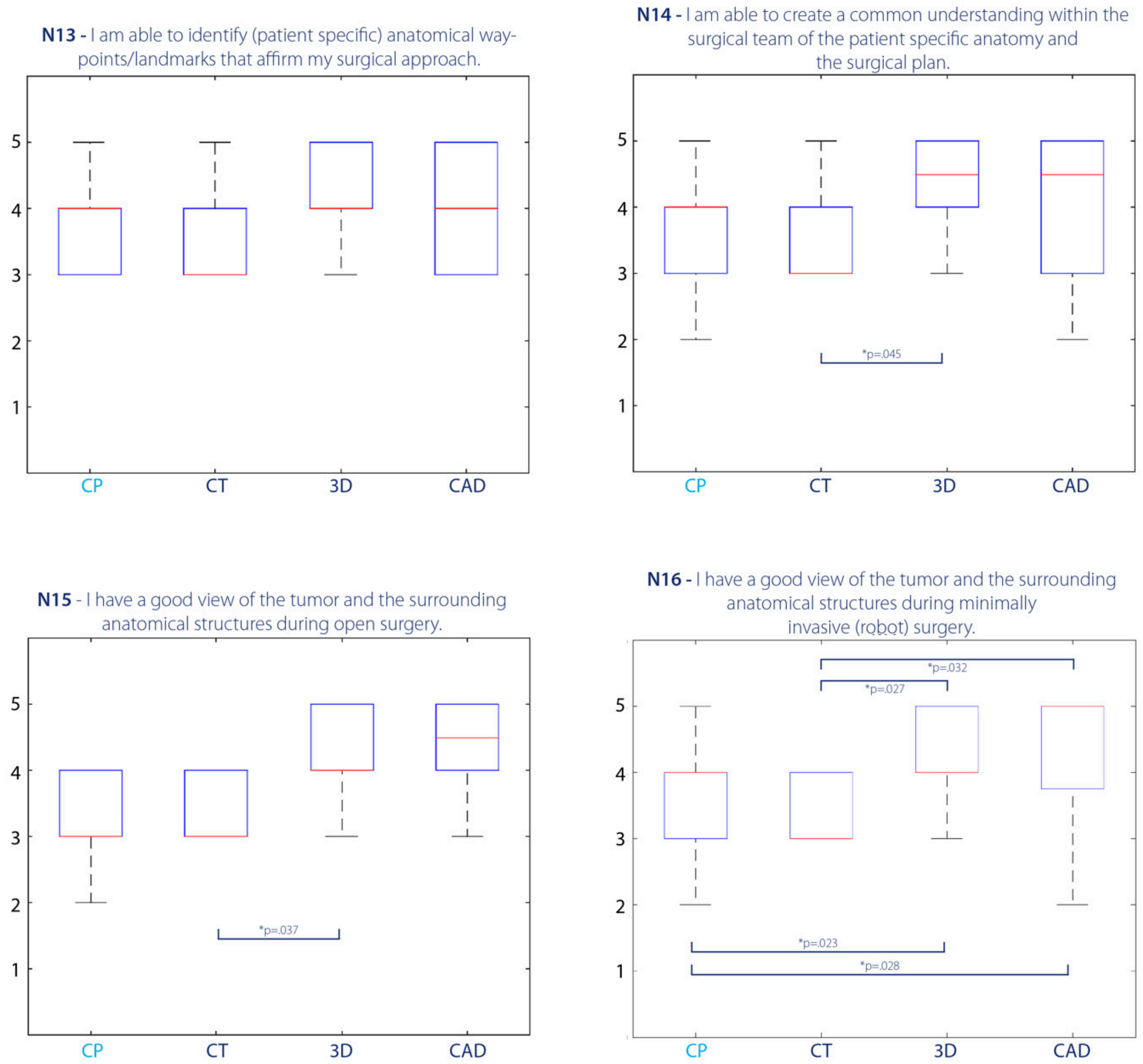

Figure S3. Box plots regarding perceived fulfilment of clinical needs (Likert scale); CP = current practice (pre-test); CT = computed tomography group; 3D = 3D group; CAD = Quantifications group. Red lines = medians; blue boxes = 25th and 75th percentile; red crosses = outlier values; dotted black line = range of values. *p < .05; **p<.001.

Supplementary material S5 – Prediction accuracy for determining an anatomical variation

Table S1. Prediction accuracy for determining an anatomical variation.

Category	CT-group Correct (n)/total (n)	3D-group Correct (n)/total (n)	CAD-group Correct (n)/total (n)
**All cases combined**	22/27	25/28	26/29
Cases with arterial variation	14/18	17/19	16/19
Cases without arterial variation	8/9	8/9	10/10

Prediction accuracy was calculated by the formula: (number of correct predictions) / (total number of predictions); Correct (n) = correct predictions; Total (n) = total number of predictions.

Supplementary material S6 – Confidence levels

Table S2. P-values regarding the confidence levels of radiologically resectable compared to radiologically borderline resectable cases.

Category	CT-condition p-value	3D-condition p-value	CAD-condition p-value
Resectability	0.0054*	<0.001*	0.029*
Vascular resection	0.0086*	<0.001*	0.0069*

*p-value < 0.05.
